# Supplementary material for: Developing interconnectedness is critical in retaining rural general practitioners: A qualitative thematic analysis of recently recruited general practitioners to South East New South Wales, Australia
Source: PLOS Glob Public Health. 2024 Mar 18;4(3):e0002860. doi: 10.1371/journal.pgph.0002860 (PMC10947638; doi:10.1371/journal.pgph.0002860)
Supplement: S1 Annex — (DOCX) [file pgph.0002860.s001.docx]

**Annex 1:**

Table 1: Semi-structured interview questions

| 1 | Basic demographic details   - Age/ gender/ rural origin/ current position/bonding – when appointed. |
| --- | --- |
| 2 | Tell me a little about how your journey in becoming a rural doctor?   - Clarify details: high school, undergraduate/ postgraduate study, rural clinical school, rural speciality training - Were there specific points where you reconsidered whether to work rurally? |
| 3 | Who or what influenced your decision to work rurally at these points?   - How? - Did these influences change as your career progressed? |
| 4 | How did your training influence your decision to work rurally?   - Can you recall specific events/experiences in your work or education which either positively or negatively influenced you? - Is there anything we could do with your education to encourage more doctors to work rurally? |
| 5 | What brought you to work in your current practice?   - Specialty interests |
| 6 | How has the type of supervision you have had prepared you for rural practice? |
| 7 | What aspects of your workplaces have you found positive or negative?   - What aspects of the practice influence or have influenced your decision to practice rurally? |
| 8 | Where do you see your career heading?   - Why? - What would improve your work satisfaction in your current job? |
