# Supplementary material for: Developing interconnectedness is critical in retaining rural general practitioners: A qualitative thematic analysis of recently recruited general practitioners to South East New South Wales, Australia
Source: PLOS Glob Public Health. 2024 Mar 18;4(3):e0002860. doi: 10.1371/journal.pgph.0002860 (PMC10947638; doi:10.1371/journal.pgph.0002860)
Supplement: Attachment — Submitted filename: Response to Reviewers.docx [file pgph.0002860.s002.docx]

Suzanne Bain-Donohue

School of Medicine and Psychology

College of Health and Medicine

Australian National University

54 Mills Road

ACTON  ACT 2601

M: 61 0403 022 593

E: [suzanne.bain-donohue@anu.edu.au](mailto:suzanne.bain-donohue@anu.edu.au)

14^th^ December 2023

Henry Zakumumpa, PhD
Academic Editor
PLOS Global Public Health

c/- [em@editorialmanager.com](mailto:em@editorialmanager.com)

Dear Dr Zakumumpa

**RE: PLOS Global Public Health Decision: Revision required [PGPH-D-23-01460] - EMID:cf55a15c1d4c699d]**

On behalf of the authors of our study, I would like to thank you and the reviewers for your consideration and feedback on our manuscript. We have considered each point raised and our response is outlined below.

| **Journal Requirements:** | **Response:** |
| --- | --- |
| 1. In the ethics statement in the Methods, you have specified that verbal consent was obtained. Please provide additional details regarding how this consent was documented and witnessed, and state whether this was approved by the IRB. | Participants all signed written consent forms once agreeing to take part in the study. Prior to commencing the interview the participants were asked to confirm their consent to participate. This was done to ensure they were still willingly volunteering to participate, as some time may have lapsed since signing the written consent form.  Changes to the manuscript (line 120-123) have been made to make this process clearer to the reader.  Line 141 – 142 indicates the ethics was received by an approved HREC. This included the approval of the consent form signed by all participants. |
| We are pleased to share comments from our reviewers. Please pay attention to each of the comments raised by the reviewers. I wish to point you to particularly comments regarding the framing of the paper and the methodology. Observations that the authors 'used thematic analysis for quantitative data' are especially problematic. | It is noted that a reviewer appears to have misinterpreted the methodology undertaken in this research.  The research we conducted was only of a qualitative nature as described from Line 117-124. None of our analysis was of quantitative data. We used qualitative and quantitative literature to inform and develop the initial codes (literature review), which were modifiable (Line 130-131). Forming codes is an essential component to analysing qualitative data. This assisted us to identify concepts already addressed in others’ research (see figure 1) and gaps in the literature. |
| 1. Does this manuscript meet PLOS Global Public Health’s [publication criteria](https://aus01.safelinks.protection.outlook.com/?url=https%3A%2F%2Fjournals.plos.org%2Fglobalpublichealth%2Fs%2Fcriteria-for-publication&data=05%7C01%7Csuzanne.bain-donohue%40anu.edu.au%7Cd1a10518da9d48b9fdba08dbce829c45%7Ce37d725cab5c46249ae5f0533e486437%7C0%7C0%7C638330832593794083%7CUnknown%7CTWFpbGZsb3d8eyJWIjoiMC4wLjAwMDAiLCJQIjoiV2luMzIiLCJBTiI6Ik1haWwiLCJXVCI6Mn0%3D%7C3000%7C%7C%7C&sdata=M2PuNCFDZVtGr9fYzYJaC5yR6mMx2FJQzjbzLKgJD1A%3D&reserved=0)? Is the manuscript technically sound, and do the data support the conclusions? The manuscript must describe methodologically and ethically rigorous research with conclusions that are appropriately drawn based on the data presented.  Reviewer #1: Yes  Reviewer #2: No | We have refined and provided additional description within the manuscript to address the methodological and ethical issues/queries identified by the reviewers. |
| 2. Has the statistical analysis been performed appropriately and rigorously?  Reviewer #1: N/A  Reviewer #2: I don't know | As this is a qualitative study only, no statistical analysis was undertaken. |
| 3. Have the authors made all data underlying the findings in their manuscript fully available (please refer to the Data Availability Statement at the start of the manuscript PDF file)? Reviewer #1: Yes  Reviewer #2: Yes | No action required. |
| 4. Is the manuscript presented in an intelligible fashion and written in standard English? Reviewer #1: Yes  Reviewer #2: Yes | No action required. |
| **Reviewer 1** |  |
| Line 78, as part of the methods, please describe the overarching research design. | Additional description has been included from Line 79-81. |
| Line 82, 'we invited ALL doctors', specify how many. | The exact numbers working within the 60 registered practices we contacted was unknown. However all doctors working within the 60 practices (and had not been working in the region >10 years) were invited to participate.  Refinement of description has been included in manuscript (line 84). |
| Line 82, 'excluding these', specify how many. | Unknown. The exact numbers working in each practice and at what FTE capacity is not known. |
| Table 1, Perhaps just mention the broad categories in the text, and move this table to annex. | Manuscript has been updated to reflect this suggestion (Line 129-131) |
| Line 325-335, Please focus on the dissonance aspect and move the content to any of the aforementioned appropriate themes. For now it comes across as repetition/redundant. | Upon review, we agree with the reviewer and have removed this section from the manuscript. Whilst it makes an important point it does not fit directly into the theme being described in this section. |
| Line 137, 'meetings aimed at developing a conceptual framework', I think it is worth sharing a visual of the framework as opposed to the above table with the interview instrument. | The table identified has been moved to an annex with the information contained summarised as per above (Line 129-131). The authors believe representing the framework visually will not allow for the readers understanding of the dynamic nature of the process. |
| The study seems to draw on discourses of social capital, judging from the focus in framing the findings around interconnectedness across three domains. Perhaps presenting explicitly the underlying framework along with sufficient description can make the paper richer. | The manuscript has been modified to explicitly identify the influence of social capital discourse and the positioning of the underlying theoretical framework in the findings (Line 395) |
| The use of theory is also warranted (e.g. social capital or personality related) to shed further light on the findings about the role of the three interconnectedness domains. | The manuscript has been modified to identify the influence of a theoretical framework. (Line 395, 404-406) |
| I find the notion of 'doctors interconnectedness' a bit vague perhaps due to lack of a theoretical grounding. Picking on your analysis about dissonance in expectation and actual experience, I wonder if interconnectedness can be rephrased as 'alignment in expectation and experience of doctors across the three domains'? | Manuscript has been modified to further clarify the authors’ argument of interconnectedness (line 395-396).  A definition of connectedness used in this study has been added to the manuscript to provide greater clarification to the reader. |
| The findings suggest that retention is contingent on personal, professional, OR geographic interconnectedness, with some differentiated patterns between the two groups of doctors. Most importantly decision to stay or leave seem to be mediated by personality or personal circumstances. Related studies seem to underscore the significance of personality. See two related sources for your reference or integration: Rural temperament and character: A new perspective on retention of rural doctors, by Eley, Young, Shrapnel Is personality the missing link in understanding recruitment and retention of rural general practitioners? by Jones and Humphreys | The authors acknowledge that personality and personal circumstance play a part in the recruitment and retention of doctors (Line 77-78). This element of rural workforce recruitment/retention has been well documented (see line 48-50). We have consciously steered away from these traits in this paper as the domains of personal, professional and geographic connectedness emerged through the analysis process of this study and subsequently identifies information regarding rural workforces retention that very little has been written about. The authors feel that incorporating the reviewers suggested elements into the paper will detracted from the arguments raised and will not be supported by the data we collected for this study (as it was not our intention to collect such data). |
| **Reviewer 2** |  |
| The authors have written a paper on a topic that is of interest to them which comes across in the article which needs to be reframed to provide an academic contribution. For example, the authors say “Axiologically, the team shared an explicit interest in understanding to increase the retention of doctors working in rural areas and this was articulated in the written participant information sheets we provided.” The article would be improved if the authors reframed the paper for the reader, e.g. The aim of the research was to … and then provided academic reasoning for the need for the research they have conducted. To this point, the gap in the literature to which this study contributes needs to be presented clearly prior to the methods section. | Reference to our own interest in this research has been removed, including the description of the authors and their attributes.  The Introduction has been refined and the identification of gaps in the literature has now been made more apparent to the reader.  The introduction now incorporates the aims of the research, academic reasoning/ workforce need for this research. |
| The methods also need to be revised. The participant section needs more detailed information, e.g. how did you identify the potential sample for your study? The participant information is hard to follow written in this way, please consider putting it into a table. Then, the quotes make more sense to the reader, e.g. is the quote from a rural doctor who has been there for 1 year or 7 years? The authors had an interest in retention yet they excluded those that been there more than 10 years – why? The exclusion criteria needs to be explained. Also the section about the researchers feels unnecessary. The authors say that they sent a participant information sheet but then say they had verbal consent to participate in the study. Was verbal consent approved by the HREC, this seems inconsistent with usual practice in Australian universities. | The Methods section of the manuscript has been revised taking into account the questions and suggestions raised by the reviewer.  Noting:   - The period of service in a location is not differentiated. - The exclusion criteria has now been explained. - The Researcher section has been deleted. - The consent question has been addressed.   Additionally, we believe that reconfirming consent is a good practice, as it allows ongoing communication between the participant and the researchers regarding the voluntary nature of participation and the ongoing decision for the participant to stay involved. This also helps reduce the risk of participants withdrawing their consent further down the line. |
| In terms of data collection, a reflexive thematic analysis was conducted on quantitative and qualitative data. It is unusual to use a qualitative data analysis technique to analyze quantitative data. The data was initially coded by hand and then coded with NVIVO. It is unclear why this was done in this way. The methodology needs further explanation with references to support the chosen data analysis techniques chosen for their data. | The research we conducted was only of a qualitative nature as described from Line 117-124. None of our analysis was of quantitative data. We used qualitative and quantitative literature to inform and develop the initial codes, which were modifiable (Line 130-131). Forming codes is an essential component to analysing qualitative data. This assisted us to identify concepts already addressed in others’ research (see figure 1) and gaps in the literature.  Our methodology of initially using hand coding and then coding with NVivo sought to utilise the advantages of both methods. Namely, Hand coding allows researchers to develop an intimate understanding of the data. Reading and manually coding each piece of information can lead to a more profound connection with the content. Whereas, NVivo provides a structured and organized environment for coding. The software allows researchers to create hierarchies of codes, link codes to themes, and visualize relationships, facilitating a more organized analysis. Furthermore; dual coding using NVivo promotes consistency in coding, reducing the risk of human error. The software ensures that codes are consistently applied according to predefined criteria.  Additional description of the reasoning of the methodology and reference has been added to the manuscript (Line 132 - 133). |
| Of note, there are a lot of comparisons between NTDs and IMDs, this appears important to the findings but not positioned in the frontend of the article as being purposely investigated. | The recruitment of participants did not specifically seek to draw on the two groups of doctors, it was therefore not positioned within the front end of the article. Whilst we recognised that there were two distinct groups within the research, we did not predict the findings of the research would indicate the extent of the differences. The emergence of the findings through the analysis is novel for this field and provides a nuanced insight into this specific workforce. The comparisons highlight the differences and support our argument that different strategies are required to retain both groups of doctors in rural settings. These findings are resultant of reflexive thematic analysis methodology utilised in this study. |

We trust that our responses to issues identified by the reviewers and the editorial team, and the revision of our manuscript meet the requirements for publication. Should you require additional information or clarification, please do not hesitate to contact me.

Suzanne Bain-Donohue

(corresponding author)
